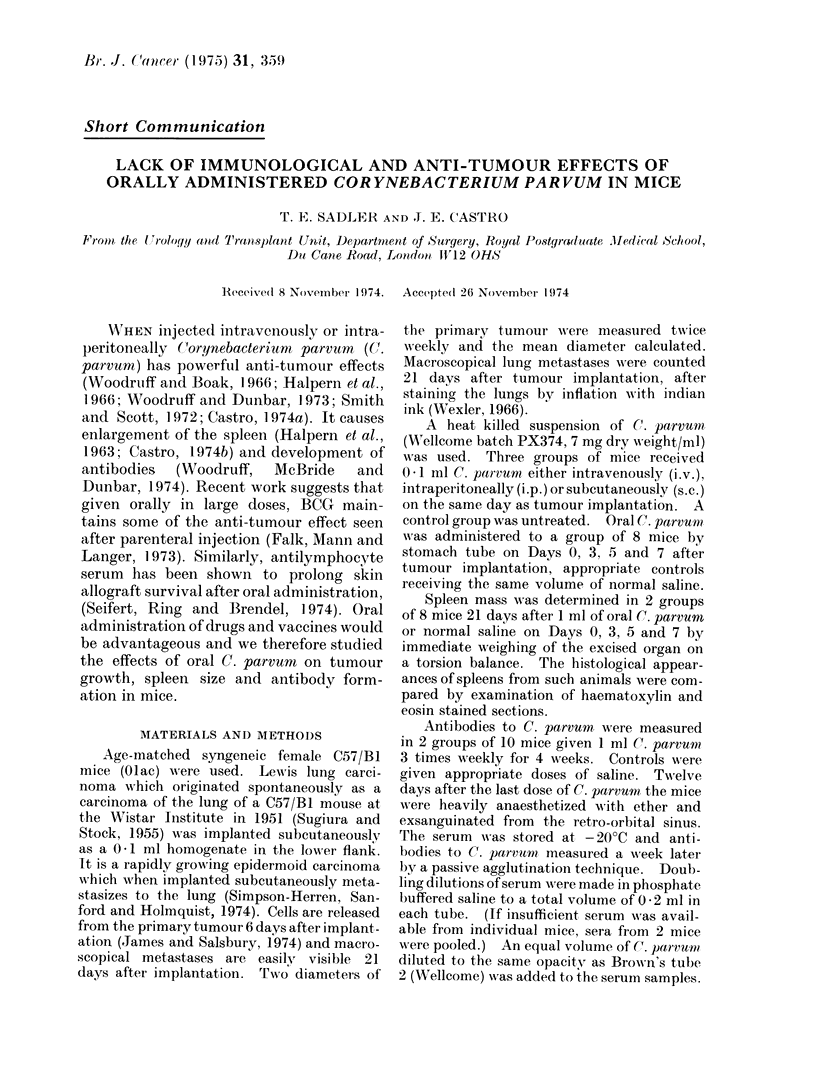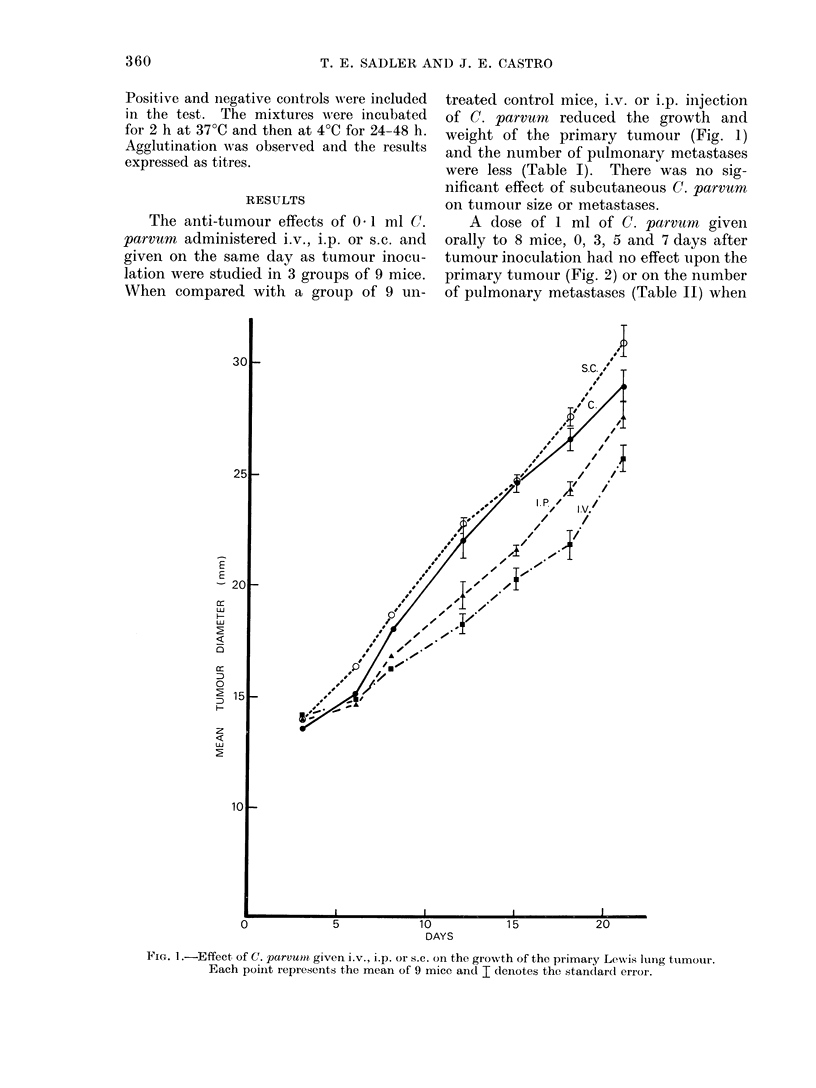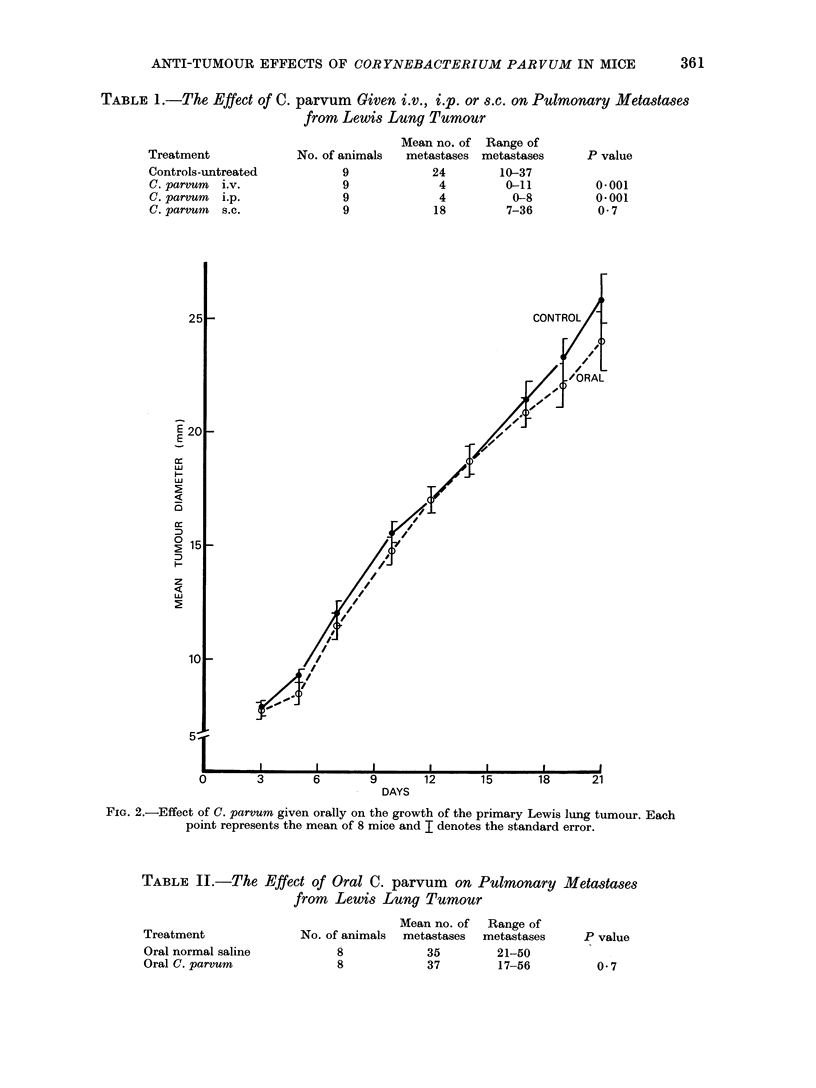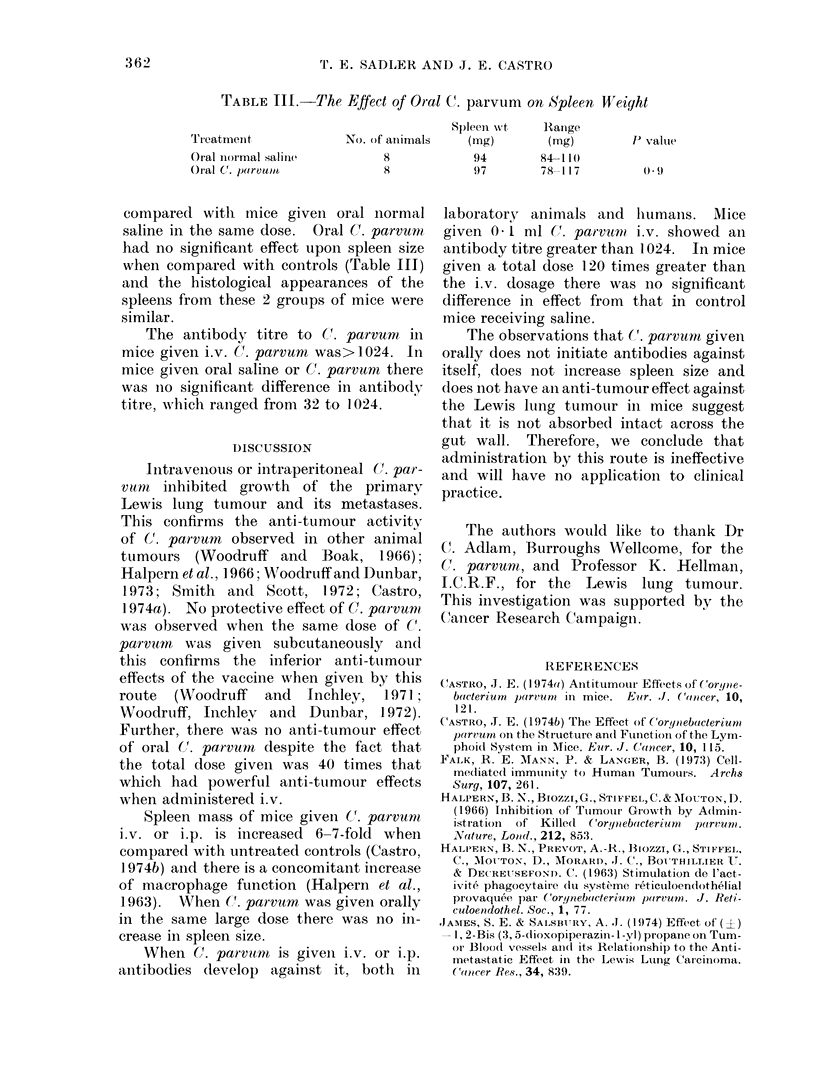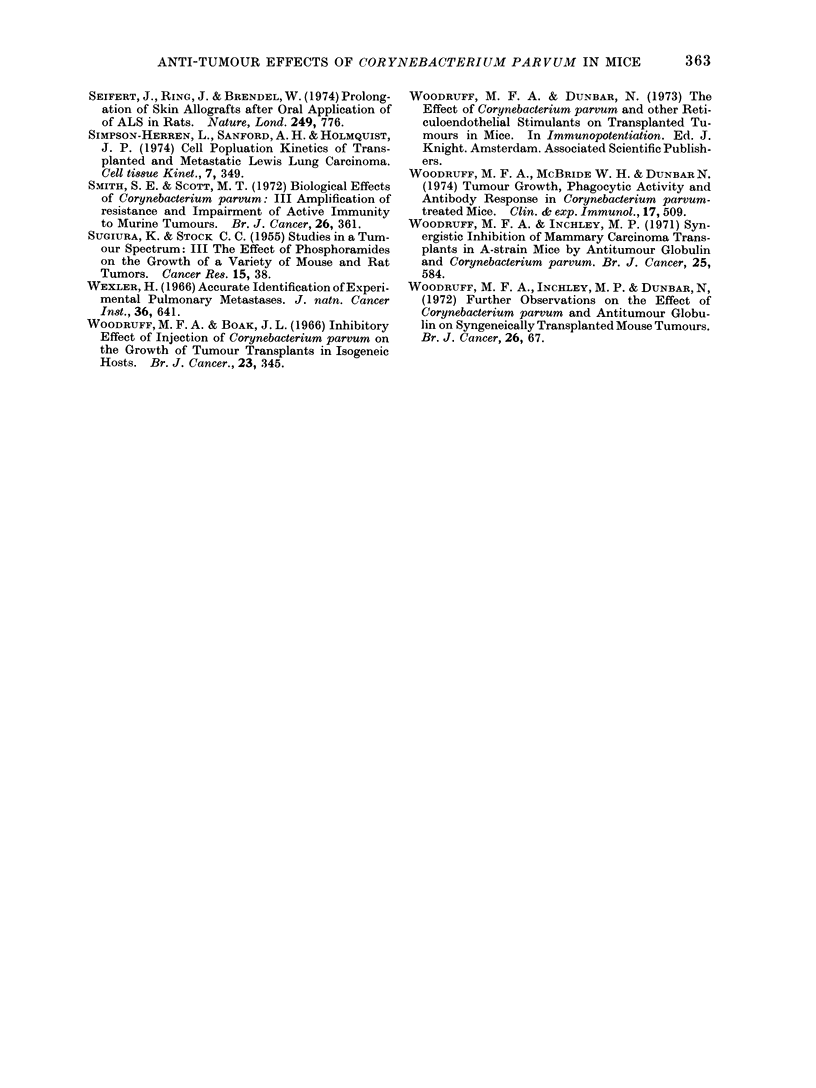# Lack of immunological and anti-tumour effects of orally administered Corynebacterium papvum in mice.

**DOI:** 10.1038/bjc.1975.71

**Published:** 1975-03

**Authors:** T. E. Sadler, J. E. Castro


*Br. J. Cancer* (1975) **31**, 359

***Short Communication***

### LACK OF IMMUNOLOGICAL AND ANTI-TUMOUR EFFECTS OF ORALLY ADMINISTERED *CORYNEBACTERIUM PARVUM* IN MICE

T. E. SADLER AND J. E. CASTRO

*From the Urology and Transplant Unit, Department of Surgery, Royal Postgraduate Medical School, Du Cane Road, London W12 OHS*

Received 8 November 1974. Accepted 26 November 1974

WHEN injected intravenously or intraperitoneally *Corynebacterium parvum* (*C. parvum*) has powerful anti-tumour effects (Woodruff and Boak, 1966; Halpern *et al.*, 1966; Woodruff and Dunbar, 1973; Smith and Scott, 1972; Castro, 1974*a*). It causes enlargement of the spleen (Halpern *et al.*, 1963; Castro, 1974*b*) and development of antibodies (Woodruff, McBride and Dunbar, 1974). Recent work suggests that given orally in large doses, BCG maintains some of the anti-tumour effect seen after parenteral injection (Falk, Mann and Langer, 1973). Similarly, antilymphocyte serum has been shown to prolong skin allograft survival after oral administration, (Seifert, Ring and Brendel, 1974). Oral administration of drugs and vaccines would be advantageous and we therefore studied the effects of oral *C. parvum* on tumour growth, spleen size and antibody formation in mice.

### MATERIALS AND METHODS

Age-matched syngeneic female C57/B1 mice (01ac) were used. Lewis lung carcinoma which originated spontaneously as a carcinoma of the lung of a C57/B1 mouse at the Wistar Institute in 1951 (Sugiura and Stock, 1955) was implanted subcutaneously as a 0·1 ml homogenate in the lower flank. It is a rapidly growing epidermoid carcinoma which when implanted subcutaneously metastasizes to the lung (Simpson-Herren, Sanford and Holmquist, 1974). Cells are released from the primary tumour 6 days after implantation (James and Salsbury, 1974) and macroscopical metastases are easily visible 21 days after implantation. Two diameters of the primary tumour were measured twice weekly and the mean diameter calculated. Macroscopical lung metastases were counted 21 days after tumour implantation, after staining the lungs by inflation with indian ink (Wexler, 1966).

A heat killed suspension of *C. parvum* (Wellcome batch PX374, 7 mg dry weight/ml) was used. Three groups of mice received 0·1 ml *C. parvum* either intravenously (i.v.), intraperitoneally (i.p.) or subcutaneously (s.c.) on the same day as tumour implantation. A control group was untreated. Oral *C. parvum* was administered to a group of 8 mice by stomach tube on Days 0, 3, 5 and 7 after tumour implantation, appropriate controls receiving the same volume of normal saline.

Spleen mass was determined in 2 groups of 8 mice 21 days after 1 ml of oral *C. parvum* or normal saline on Days 0, 3, 5 and 7 by immediate weighing of the excised organ on a torsion balance. The histological appearances of spleens from such animals were compared by examination of haematoxylin and eosin stained sections.

Antibodies to *C. parvum* were measured in 2 groups of 10 mice given 1 ml *C. parvum* 3 times weekly for 4 weeks. Controls were given appropriate doses of saline. Twelve days after the last dose of *C. parvum* the mice were heavily anaesthetized with ether and exsanguinated from the retro-orbital sinus. The serum was stored at −20°C and antibodies to *C. parvum* measured a week later by a passive agglutination technique. Doubling dilutions of serum were made in phosphate buffered saline to a total volume of 0·2 ml in each tube. (If insufficient serum was available from individual mice, sera from 2 mice were pooled.) An equal volume of *C. parvum* diluted to the same opacity as Brown's tube 2 (Wellcome) was added to the serum samples.

Positive and negative controls were included in the test. The mixtures were incubated for 2 h at 37°C and then at 4°C for 24–48 h. Agglutination was observed and the results expressed as titres.

### RESULTS

The anti-tumour effects of 0·1 ml *C. parvum* administered i.v., i.p. or s.c. and given on the same day as tumour inoculation were studied in 3 groups of 9 mice. When compared with a group of 9 untreated control mice, i.v. or i.p. injection of *C. parvum* reduced the growth and weight of the primary tumour (Fig. 1) and the number of pulmonary metastases were less (Table I). There was no significant effect of subcutaneous *C. parvum* on tumour size or metastases.

A dose of 1 ml of *C. parvum* given orally to 8 mice, 0, 3, 5 and 7 days after tumour inoculation had no effect upon the primary tumour (Fig. 2) or on the number of pulmonary metastases (Table II) when

FIG. 1.—Effect of *C. parvum* given i.v., i.p. or s.c. on the growth of the primary Lewis lung tumour. Each point represents the mean of 9 mice and ⌶ denotes the standard error.

TABLE I.—*The Effect of* C. parvum *Given i.v., i.p. or s.c. on Pulmonary Metastases from Lewis Lung Tumour*

| Treatment | No. of animals | Mean no. of metastases | Range of metastases | *P* value |
|---|---|---|---|---|
| Controls-untreated | 9 | 24 | 10–37 | |
| *C. parvum* i.v. | 9 | 4 | 0–11 | 0·001 |
| *C. parvum* i.p. | 9 | 4 | 0–8 | 0·001 |
| *C. parvum* s.c. | 9 | 18 | 7–36 | 0·7 |

FIG. 2.—Effect of *C. parvum* given orally on the growth of the primary Lewis lung tumour. Each point represents the mean of 8 mice and I denotes the standard error.

TABLE II.—*The Effect of Oral* C. parvum *on Pulmonary Metastases from Lewis Lung Tumour*

| Treatment | No. of animals | Mean no. of metastases | Range of metastases | *P* value |
|---|---|---|---|---|
| Oral normal saline | 8 | 35 | 21–50 | |
| Oral *C. parvum* | 8 | 37 | 17–56 | 0·7 |

TABLE III.—*The Effect of Oral* C. parvum *on Spleen Weight*

| Treatment | No. of animals | Spleen wt (mg) | Range (mg) | *P* value |
|---|---|---|---|---|
| Oral normal saline | 8 | 94 | 84–110 | |
| Oral *C. parvum* | 8 | 97 | 78–117 | 0·9 |

compared with mice given oral normal saline in the same dose. Oral *C. parvum* had no significant effect upon spleen size when compared with controls (Table III) and the histological appearances of the spleens from these 2 groups of mice were similar.

The antibody titre to *C. parvum* in mice given i.v. *C. parvum* was>1024. In mice given oral saline or *C. parvum* there was no significant difference in antibody titre, which ranged from 32 to 1024.

### DISCUSSION

Intravenous or intraperitoneal *C. parvum* inhibited growth of the primary Lewis lung tumour and its metastases. This confirms the anti-tumour activity of *C. parvum* observed in other animal tumours (Woodruff and Boak, 1966); Halpern *et al.*, 1966; Woodruff and Dunbar, 1973; Smith and Scott, 1972; Castro, 1974*a*). No protective effect of *C. parvum* was observed when the same dose of *C. parvum* was given subcutaneously and this confirms the inferior anti-tumour effects of the vaccine when given by this route (Woodruff and Inchley, 1971; Woodruff, Inchley and Dunbar, 1972). Further, there was no anti-tumour effect of oral *C. parvum* despite the fact that the total dose given was 40 times that which had powerful anti-tumour effects when administered i.v.

Spleen mass of mice given *C. parvum* i.v. or i.p. is increased 6–7-fold when compared with untreated controls (Castro, 1974*b*) and there is a concomitant increase of macrophage function (Halpern *et al.*, 1963). When *C. parvum* was given orally in the same large dose there was no increase in spleen size.

When *C. parvum* is given i.v. or i.p. antibodies develop against it, both in laboratory animals and humans. Mice given 0·1 ml *C. parvum* i.v. showed an antibody titre greater than 1024. In mice given a total dose 120 times greater than the i.v. dosage there was no significant difference in effect from that in control mice receiving saline.

The observations that *C. parvum* given orally does not initiate antibodies against itself, does not increase spleen size and does not have an anti-tumour effect against the Lewis lung tumour in mice suggest that it is not absorbed intact across the gut wall. Therefore, we conclude that administration by this route is ineffective and will have no application to clinical practice.

The authors would like to thank Dr C. Adlam, Burroughs Wellcome, for the *C. parvum*, and Professor K. Hellman, I.C.R.F., for the Lewis lung tumour. This investigation was supported by the Cancer Research Campaign.

### REFERENCES

CASTRO, J. E. (1974*a*) Antitumour Effects of *Corynebacterium parvum* in mice. *Eur. J. Cancer*, **10**, 121.

CASTRO, J. E. (1974*b*) The Effect of *Corynebacterium parvum* on the Structure and Function of the Lymphoid System in Mice. *Eur. J. Cancer*, **10**, 115.

FALK, R. E. MANN, P. & LANGER, B. (1973) Cell-mediated immunity to Human Tumours. *Archs Surg*, **107**, 261.

HALPERN, B. N., BIOZZI, G., STIFFEL, C. & MOUTON, D. (1966) Inhibition of Tumour Growth by Administration of Killed *Corynebacterium parvum*. *Nature, Lond.*, **212**, 853.

HALPERN, B. N., PREVOT, A.-R., BIOZZI, G., STIFFEL, C., MOUTON, D., MORARD, J. C., BOUTHILLIER U. & DECREUSEFOND. C. (1963) Stimulation de l'activité phagocytaire du système réticuloendothélial provaquée par *Corynebacterium parvum*. *J. Reticuloendothel. Soc.*, **1**, 77.

JAMES, S. E. & SALSBURY, A. J. (1974) Effect of (±) — 1, 2-Bis (3, 5-dioxopiperazin-1-yl) propane on Tumor Blood vessels and its Relationship to the Antimetastatic Effect in the Lewis Lung Carcinoma. *Cancer Res.*, **34**, 839.

SEIFERT, J., RING, J. & BRENDEL, W. (1974) Prolongation of Skin Allografts after Oral Application of of ALS in Rats. *Nature, Lond.* **249**, 776.

SIMPSON-HERREN, L., SANFORD, A. H. & HOLMQUIST, J. P. (1974) Cell Popluation Kinetics of Transplanted and Metastatic Lewis Lung Carcinoma. *Cell tissue Kinet.*, **7**, 349.

SMITH, S. E. & SCOTT, M. T. (1972) Biological Effects of *Corynebacterium parvum:* III Amplification of resistance and Impairment of Active Immunity to Murine Tumours. *Br. J. Cancer*, **26**, 361.

SUGIURA, K. & STOCK C. C. (1955) Studies in a Tumour Spectrum: III The Effect of Phosphoramides on the Growth of a Variety of Mouse and Rat Tumors. *Cancer Res.* **15**, 38.

WEXLER, H. (1966) Accurate Identification of Experimental Pulmonary Metastases. *J. natn. Cancer Inst.*, **36**, 641.

WOODRUFF, M. F. A. & BOAK, J. L. (1966) Inhibitory Effect of Injection of *Corynebacterium parvum* on the Growth of Tumour Transplants in Isogeneic Hosts. *Br. J. Cancer.*, **23**, 345.

WOODRUFF, M. F. A. & DUNBAR, N. (1973) The Effect of *Corynebacterium parvum* and other Reticuloendothelial Stimulants on Transplanted Tumours in Mice. In *Immunopotentiation*. Ed. J. Knight. Amsterdam. Associated Scientific Publishers.

WOODRUFF, M. F. A., MCBRIDE W. H. & DUNBAR N. (1974) Tumour Growth, Phagocytic Activity and Antibody Response in *Corynebacterium parvum*-treated Mice. *Clin. & exp. Immunol.*, **17**, 509.

WOODRUFF, M. F. A. & INCHLEY, M. P. (1971) Synergistic Inhibition of Mammary Carcinoma Transplants in A-strain Mice by Antitumour Globulin and *Corynebacterium parvum. Br. J. Cancer*, **25**, 584.

WOODRUFF, M. F. A., INCHLEY, M. P. & DUNBAR, N, (1972) Further Observations on the Effect of *Corynebacterium parvum* and Antitumour Globulin on Syngeneically Transplanted Mouse Tumours. *Br. J. Cancer*, **26**, 67.